# Social Support for Acculturative Stress, Job Stress, and Perceived Discrimination Among Migrant Workers Moderates COVID-19 Pandemic Depression

**DOI:** 10.3389/ijph.2022.1604643

**Published:** 2022-08-05

**Authors:** Youlim Kim, Hyeonkyeong Lee, Mikyung Lee

**Affiliations:** ^1^ College of Nursing, Kosin University, Pusan, South Korea; ^2^ College of Nursing, Yonsei University, Seoul, South Korea

**Keywords:** acculturation, acculturative stress, coronavirus, depression, occupational stress, social support, transients and migrants

## Abstract

**Objectives:** This study aimed to investigate the practical moderating effect of social support on the relationship between acculturative stress, job stress, and perceived discrimination, and depression among migrant workers during the coronavirus disease-19 pandemic as a vulnerable group susceptible to mental health problems.

**Methods:** Data for this cross-sectional descriptive study were collected using an online survey from 214 Vietnamese and Cambodian migrant workers, who are among the largest migrant groups residing in South Korea. Participants were asked to report on acculturative stress, job stress, perceived discrimination, depression, and social support through questionnaires in their native languages.

**Results:** The findings showed that acculturative stress affected depression, and this effect was moderated by social support. The impact of acculturative stress on depression was significant in the group with low mean scores of social support. However, the effect of the interaction of social support on the relationship of job stress and perceived discrimination to depression was not statistically significant.

**Conclusion:** Our findings suggest the need for differentiated strategies to improve the mental health of migrant workers based on the level of social support.

## Introduction

The coronavirus disease 2019 (COVID-19) is having a significant impact on the daily life and work of migrant workers, who are susceptible to employment insecurity, delayed decisions of legal status, and lack of culturally and linguistically accessible information about COVID-19 [1]. Migrant workers are known to be vulnerable to common mental health problems, including depression and anxiety [2, 3]. A survey of migrants in need of help from others showed that 82.7% of the respondents needed a conversation partner due to depression; thus, it can be inferred that depression is an important life problem for migrants’ health [4]. Several studies have reported that migrant workers are exposed to poor mental health due to unemployment, poor living conditions, social inequality, and discrimination [5, 6]. Among the negative mental health issues, the most reported psychological problem is depression [2]. Due to the COVID-19 pandemic, migrant workers are now at increased risk of mental health problems, such as fear, stress, anxiety, and depression [7].

Earlier studies have shown that acculturation of migrant workers was strongly related to their mental health as it affects health through healthy behavior, access to health care, social support, and acculturative stress [8–11]. The relationship was proved even in internal migrant workers who moved from rural to urban cities in China and showed lower levels of self-rated health and mental health in the marginalized group than the assimilated group [12]. In light of the particular vulnerability of migrant workers who came to Korea after being born in a country with a different culture, it would be more difficult for them to adapt to the new culture or society in the context of the sociocultural environment.

Social support has long been considered an important protective factor in mitigating adverse mental health. According to the stress-buffering model, social support may reduce adverse psychological effects on individuals’ mental health [13]. In a study of migrant workers residing in South Korea, high social support corresponded to their increased psychological well-being [14]. In addition to the direct effect of social support on mental health, perceived social support significantly moderates the effect of perceived discrimination [15], stress [16], and job stressors on depression [17].

**FIGURE 1:**
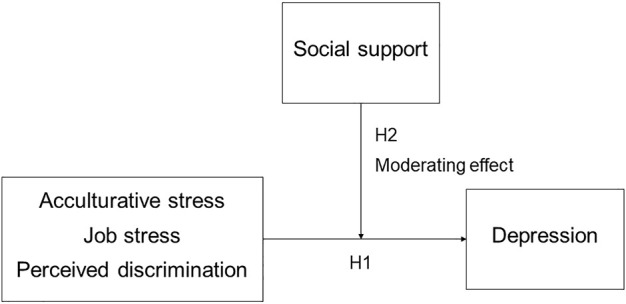
Structural model for hypothesis testing (South Korea, 2020).

The COVID-19 pandemic has adversely affected people’s mental health due to COVID-19-related health concerns, financial instability, and misinformation [18]. The number of confirmed COVID-19 cases in South Korea has risen sharply since mid-August, 2020, and on 27 August 2020, the number of new daily cases exceeded 400 for the first time in 5 months. Accordingly, since 30 August, the government implemented reinforced social distancing in the metropolitan area; the use of restaurants was prohibited after 9:00 p.m., and industrial factories limited the number of employees to minimize work density. According to a study [19] that explored the lives of migrant workers during the time of COVID-19, they experienced various difficulties such as discrimination, isolation, socioeconomic crisis, and anxiety about their lack of protection from infectious diseases due to their status as foreigners. For these reasons, the relationship between various kinds of stress experienced by migrant workers and social support during the COVID-19 pandemic needed to be identified. A review of the trends in health-related research on migrant workers in Korea showed that mental health research mainly investigated depression and job stress, with most of the studies focusing on their correlates [20]. To explore differentiation strategies for promoting the mental health of migrant workers, urgent research is needed to verify the protective effects of social support during the COVID-19 pandemic. Therefore, this study first hypothesized that perceived discrimination, job stress, and acculturative stress were predictors of migrant workers’ depression (H1). Subsequently, it was also hypothesized that social support would moderate the relationship between perceived discrimination, job stress, and acculturative stress and depression (H2) among migrant workers in South Korea (Figure 1).

## Methods

### Study Design

This study employed a cross-sectional design to examine the relationship between discrimination, acculturative stress, job stress, and depression, and the moderating effects of social support on the relationship between the determinants of depression and depression among migrant workers during the COVID-19 pandemic.

### Sample

The study participants were salaried migrant workers living in Korea and were limited to those of Vietnamese and Cambodian nationality, who were among the largest migrant groups. The inclusion criteria were those aged 18 years or older, migrant workers legally registered at the immigration office, those who read and understood questionnaires translated into their native language, those who understood the purpose of this study, and those who voluntarily consented to data collection. Using a non-proportional extraction method, we aimed to recruit 222 people, considering a dropout rate of 10%. However, to prevent time bias due to the extension of the data collection period, the data from 214 people including 132 Vietnamese workers and 82 Cambodian workers who responded within the survey period were used for the final analysis. According to the 2020 the Survey on Immigrants’ Living Conditions and Labor Force [21] in South Korea, Cambodian workers (13.7%) have the highest number with non-professional employment (E-9 Visa) with simple skills, followed by Vietnamese workers (13.5%). The number of adequate samples required for data analysis was verified by the post-hoc test for multiple regression analysis using the G*Power 3.1.9.2 software. The effect size was set to medium (*F* ^2^ = 0.15) in the regression analysis, the significance level *α* was 0.05, and the statistical power was 99.8% when the data of 214 people were analyzed based on four predictors. Therefore, it was considered that the sample size for the study was appropriate.

### Study Instrument

#### Depression

Depression was measured using the 10-item CES-D (Center for Epidemiologic Studies of Depression, Boston form) scale, derived from the 20-item CES-D scale [22]. This questionnaire queried feelings and actions over the previous week, which were then measured using the 4-point Likert scale. The cases of “I thought about it for a while,” or “I didn’t think about it for a while” were converted to “0” and the cases of “I sometimes had such an idea” to the case of “I always had such an idea” were converted to “1.” A total score of 4 or more is considered depression. CES-D was used in several studies of migrants living in Korea including those who came from Vietnam and Cambodia [23–25] with high levels of internal consistency ranged from 0.79 to 0.88. In the study by Kohout et al. [22], the Cronbach’s *α* was 0.80, while in the current study, the Cronbach’s *α* was 0.795.

#### Social Support

Social support was measured using the Multidimensional Scale of Perceived Social Support questionnaire developed by Zimet et al. [26] for college students. The questionnaire comprised 12 items, including family, friends, and other important persons, rated on a 7-point Likert scale. The higher the sum score, the higher the level of social support. In this study, reflecting the characteristics of migrant workers living alone in other countries, only eight items were measured, excluding four items about their families. To categorize levels of social support, mean scores ranging from 1 to 2.9 can be considered low support; a score of 3–5 can be considered moderate support; while a score of 5.1–7 can be considered high support [27]. In the original scale, Cronbach’s *α* was 0.85 for family members, 0.75 for friends, and 0.72 for other important persons, while in the current study, Cronbach’s *α* was 0.895.

#### Acculturation Stress

Acculturation stress was measured using the scale modified by Alderete et al. [28]. The questionnaire comprised 13 items, including discrimination experience (four items), language conflict (three items), and legal status (six items), rated on a 5-point Likert scale. The higher the sum score, the higher the acculturative stress. In the original scale, Cronbach’s *α* was 0.70 for discrimination experience, 0.65 for language conflict, and 0.79 for legal status. In this study, Cronbach’s *α* was 0.79 for discrimination experience, 0.81 for language conflict, and 0.84 for legal status.

#### Job Stress

Job stress was measured using the General Work Stress Scale developed by De Bruin [29]. The questionnaire comprised nine items, including the emotional, cognitive, motivational, and social impact of the interaction between the perceived needs of the individual and the workplace, rated on a 5-point Likert scale. The higher the sum score, the higher the job stress. Cronbach’s *α* of the original scale was 0.989, while that of the present study was 0.903.

#### Perceived Discrimination

Perceived discrimination was measured using a scale modified by Sanchez and Brock [30]. The questionnaire comprised 10 items rated on a 5-point Likert scale. The higher the sum score, the higher the level of perceived discrimination. In the study by Sanchez and Brock [30], Cronbach’s *α* was 0.87, while in the current study, the Cronbach’s *α* was 0.842.

#### General Characteristics

Participants provided general characteristics, such as age, gender, job, education, marital status, and income.

### Study Process

#### Translation Process

Before starting the study, we obtained permission to use and translate the tools from the original author. The translation process followed the WHO process of translation [31] and adaptation of instruments; 1) Forward translation, 2) Expert panel back-translation, 3) Pre-testing and cognitive interviewing, and 4) Final version. The researchers fluent in English and the translation languages translated the original English questionnaires into each native language (Vietnamese, Khmer). The translated questionnaires were then translated back into English by other translators fluent in English and the native languages. Mutual independence was maintained between the forward and back translators in the translation process. The research team and all translators from each country discussed the translation accuracy and agreed on the final translation version.

#### Cognitive Interview and Pre-test

A cognitive interview [32] was conducted with five Vietnamese and five Cambodian workers to check whether the contents were understood, and the questionnaires’ difficulty, suitability, and understanding were assessed. The revised version was finalized with the Vietnamese and Cambodian counselors working at the Korean Foreign Workers Support Center, which completed the translation of the questionnaire.

### Data Analysis

Statistical analysis was conducted using SPSS software version 25.0 (IBM, Chicago, IL, United States). Descriptive statistics (frequency, percentage, means, and standard deviations) were used to describe participants’ general characteristics, and Pearson correlation analysis was used to measure the correlation between variables. With depression as the dependent variable, 0 was coded as “no depression” and 1 as “depression.” Since the dependent variable, depression was binary, the values of the regression coefficients were expressed in the log odds (logit). The moderating effect of social support on the relationship between acculturation stress, job stress, and perceived discrimination and depression was analyzed by SPSS PROCESS Macro v3.4.1. (model 1, IBM) [33]. In addition, the Johnson–Neyman technique was used to confirm the areas with significant moderation effects of social support. The Johnson–Neyman technique identifies areas in which the influence of the independent variable on the dependent variable is statistically significant according to the moderator variable. Hayes [34] reported that the mean centering was not related to the reduction of multicollinearity; thus, we did not apply it in this study. The reliability of the variables was calculated as Cronbach’s *α* value, and statistical significance was set at 0.05.

### Ethical Approval

This study was approved by the Institutional Review Board of Yonsei University Health System (Y-2018-0148). Informed consent was obtained from all participants after the researcher explained the modalities of the research study. Participants’ identities were anonymous, and participants could withdraw from the survey at any time without justification.

### Data Collection

Data were collected from June 2020 to December 2020 using non-probability sampling. The questionnaire was translated and provided in the native languages to make it easier for Vietnamese and Cambodian workers to understand, and an incentive was provided after the survey to prevent missing data. For offline data collection, we placed the questionnaires at community facilities where migrant workers usually visit, such as the Korea Foreign Workers Support Center. The snowball method of posting recruitment notices to colleagues, friends, and acquaintances of migrant workers through Social Network Services (SNS) was used for online data collection. In addition, we provided a link to the survey to a church priest and an industrial manager who interacted with many migrant workers. The first page of the online survey had a link to the questionnaire, while the leaflets had a QR CODE. The participants could access details about the study content, such as the study title, purpose, and method, and fill out the consent form. To prevent non-response items in the questionnaire, all the questions required answers for the submission to be accepted. As a result, data from a total of 214 participants were valid and were used for analysis.

## Results

### Characteristics of Participants

**FIGURE 2:**
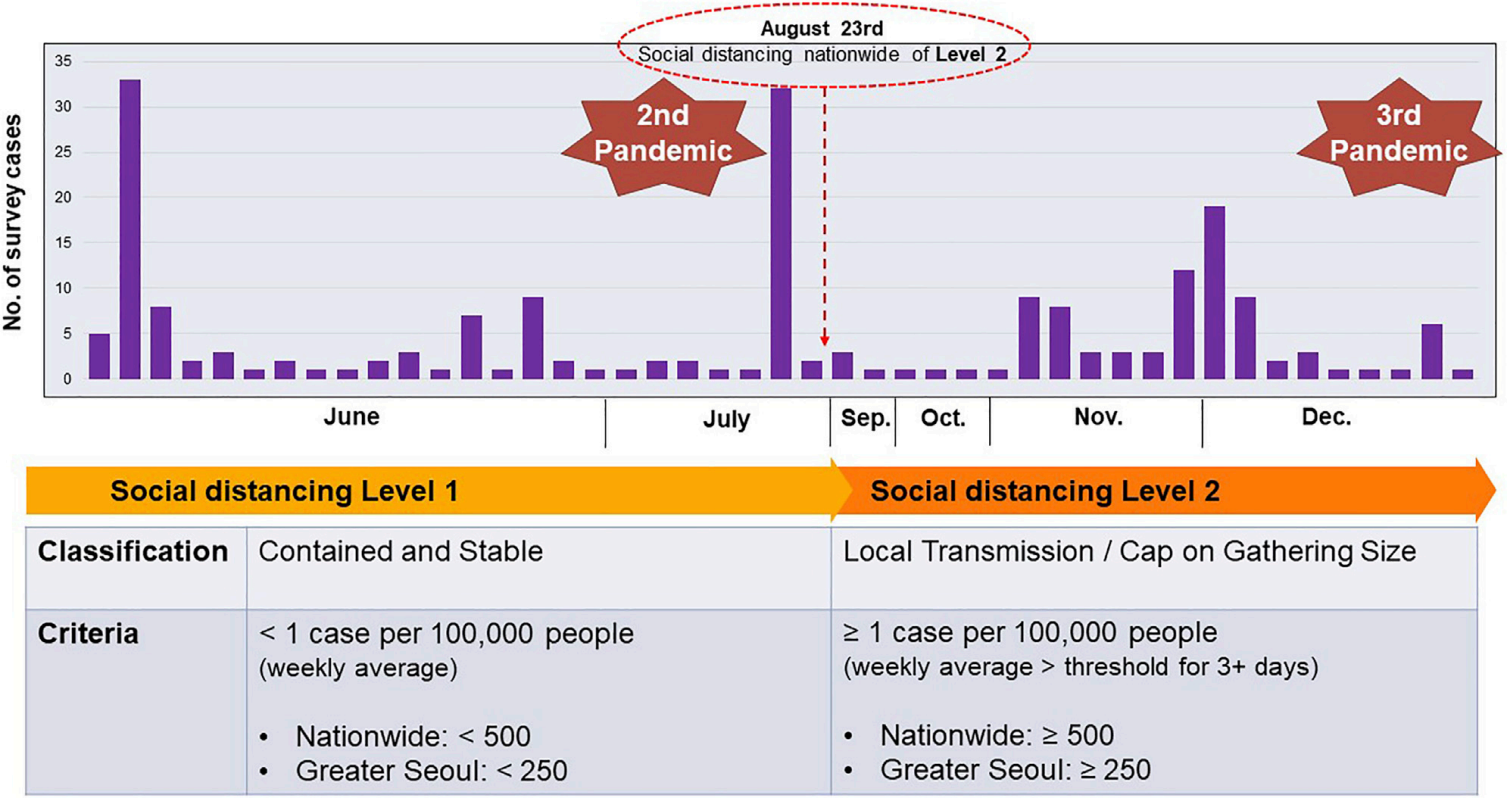
Current status of data collection in the time of Coronavirus disease-19 (South Korea, 2020).

The participants comprised 214 migrant workers, including 132 (61.7%) Vietnamese and 82 (38.3%) Cambodian workers. The male proportion of the survey respondents was 44.4%, and the average age was 30.1 ± 5.41 years. The number of COVID-19 patients in the residential areas of the study participants (metropolitan area of Gyeonggi Province and Seoul city) has increased sharply since mid-August 2020, resulting in the strengthening of social distancing measures (Figure 2). After collecting data from 91 participants, 64 workers were found to have depressive symptoms according to the CES-D scale. More details about the participants are presented in Table 1.

**TABLE 1:** General characteristics of participants (*n* = 214) (South Korea, 2020).

Variables	Mean ± SD	*n* (%)
Nationality		
Vietnam		132 (61.7%)
Cambodia		82 (38.3%)
Gender		
Male		95 (44.4%)
Age	30.1 ± 5.41	
Marital status		
Non-married		131 (61.2%)
Married		75 (35.0%)
Others (divorce)		8 (3.8%)
Education level		
≥College		163 (62.1%)
Job		
Simple labor		92 (43.0%)
Skilled labor		28 (13.1%)
Service or specialist	18 (8.4%)	
Others (farming, etc.)	76 (35.5%)	
Length of stay		
less than 5 years		144 (67.3%)
Income (USD)	1,508.89 ± 707.34	
Motivation of migration		
Family livelihood		82 (38.3%)
Skilled labor		44 (20.6%)
Culture experience		42 (19.6%)
Others (Business preparation etc.)		46 (21.5%)
Smoking		
Smoking cessation		189 (88.3%)
Past smoking		20 (9.3%)
Current smoking		5 (2.4%)
Drinking		
None		111 (51.9%)
1 time/month		74 (34.6%)
More than 2 times/month		29 (13.6%)
Depression		
Yes		64 (29.9%)

### Correlation Between Major Variables

Acculturation stress was negatively correlated with social support (*r* = −0.173) and positively correlated with job stress (*r* = 0.523), perceived stress (*r* = 0.488) and depression (*r* = 0.347). Social support was negatively correlated with depression (*r* = −0.199) but not significantly correlated with job stress and perceived stress. Table 2 presents the descriptive statistics and correlation analysis results of the major variables.

**TABLE 2:** Descriptive and correlation for major variables (South Korea, 2020).

	Range	Mean ± SD^b^	Acculturation stress	Job stress	Perceived discrimination	Social support	Depression
Acculturation stress	13–65	31.41 ± 9.29	1				
Job stress	1–5	2.78 ± 0.76	0.523**^a^	1			
Perceived discrimination	10–50	32.54 ± 6.87	0.488**^a^	0.344**^a^	1		
Social support	1–7	5.21 ± 0.99	−0.173*^a^	−0.106	−0.088	1	
Depression	—	—	0.347**^a^	0.330^**^ ^a^	0.323**^a^	−0.199**^a^	1

a *p < 0.05. **p < 0.01.

b SD, standard deviation.

### The Moderating Effect of Social Support on the Relationships Between Cultural Adaptation Stress, Job Stress, Perceived Discrimination and Depression

**FIGURE 3:**
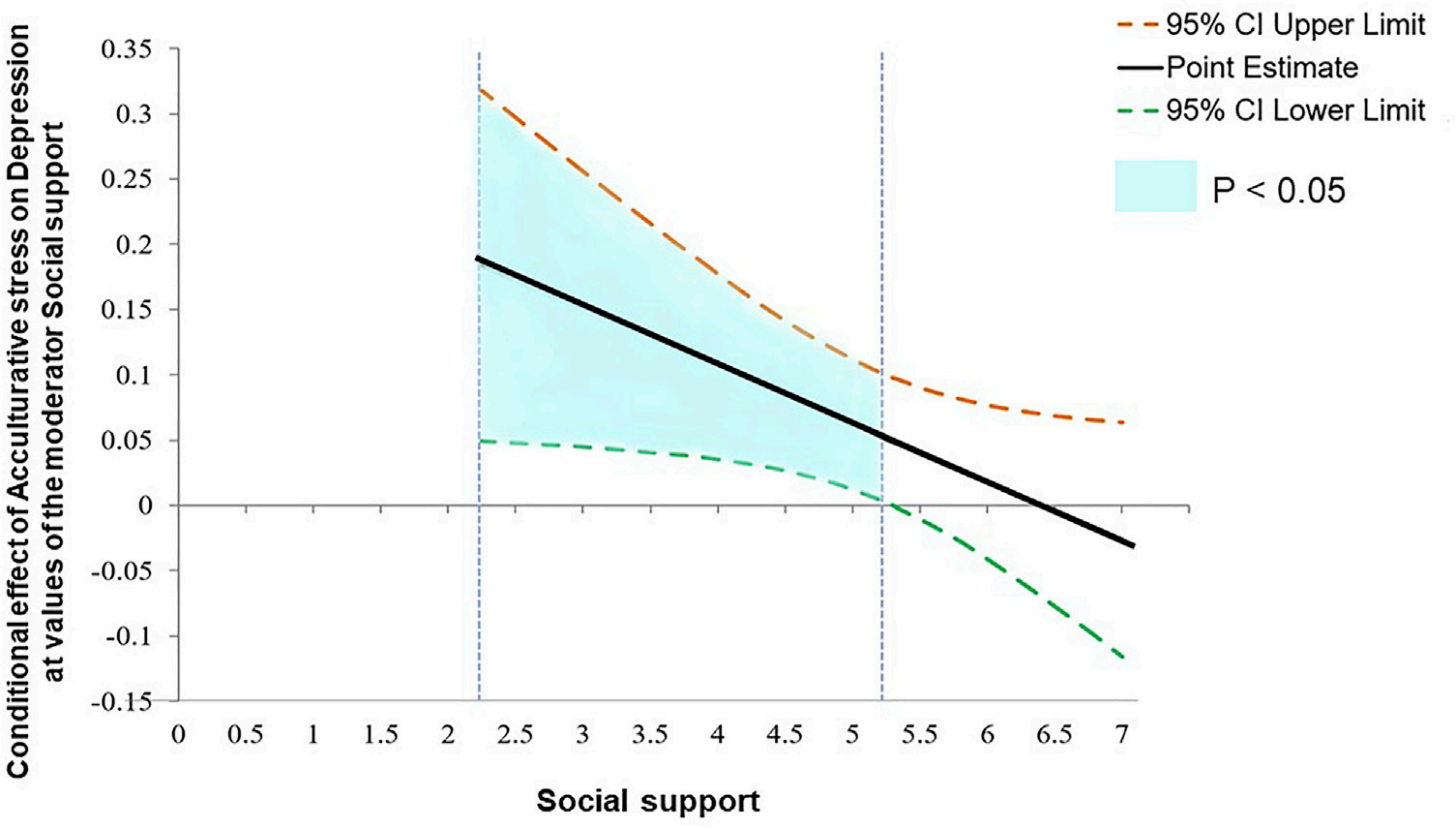
The influence of interaction between acculturation stress and social support on depression by Johnson-Neyman technique (South Korea, 2020).

Table 3 shows the results of the moderating effect of social support. The higher the acculturation stress and job stress, the higher the risk of depression (acculturative stress: log odds = −0.283, *p* = 0.014, job stress: log odds = −0.838, *p* = 0.005) in Model 1. However, perceived discrimination and social support were not significant (perceived discrimination: log odds = −0.061, *p* = 0.055, social support: log odds = −1.076, *p* = 0.135). The model fit was significant when social support was imputed as a moderator variable (Chi-square = 4.424, *p* = 0.035). The interaction effect of acculturative stress and social support was statistically significant (log odds = 0.044, *p* = 0.040). Using the Johnson–Neyman technique, significant areas were identified based on the score of 5.294 for social support. In the group whose social support was lower than 5.294, the influence of acculturative stress on depression was statistically significant, but when the social support score was higher than 5.294, the effect of acculturative stress on depression was not statistically significant. In the significant area of the Johnson–Neyman technique (the area where social support was lower than 5.294, below 49.07%), social support played a role in reducing the static effect of acculturative stress on depression. The detailed results are shown in Figure 3. The interaction effect of job stress and social support on depression was not statistically significant (log odds = 0.255, *p* = 0.352) in Model 2. Similarly, the interaction effect of perceived discrimination and social support on depression was not statistically significant (log odds = 0.005, *p* = 0.861) in Model 3.

**TABLE 3:** Testing for the moderation effect of social support on the relationship between acculturative stress, job stress, and perceived discrimination and depression (South Korea, 2020).

Variables^a^	Log odds^b^	SE^d^	Z	*p*	LLCI^d^	ULCI^d^
Model 1: Acculturation stress * Social support^c^						
Acculturative stress	-0.283	0.115	2.464	0.014	−0.508	−0.058
Job stress	−0.838	0.297	2.824	0.005	−1.420	−0.256
Perceived discrimination	−0.061	0.032	1.920	0.055	−0.124	0.001
Social support	−1.076	0.720	1.493	0.135	−2.487	0.336
Acculturative stress * Social support	0.044	0.022	−2.057	0.040	0.002	0.086
Model fit: Chi-square = 4.424, *p* = 0.035						
Model 2: Job stress * Social support^c^						
Job stress	−0.572	1.472	0.401	0.689	−3.369	2.225
Acculturative stress	0.057	0.025	2.335	0.012	0.009	0.105
Perceived discrimination	0.058	0.031	−1.896	0.058	−0.002	0.119
Social support	−1.117	0.829	1.348	0.178	−2.742	0.507
Job stress * Social support	0.255	0.274	0.931	0.352	−0.282	0.792
Model fit: Chi-square = 0.8774, *p* = 0.349						
Model 3: Perceived discrimination * Social support^c^						
Perceived discrimination	0.033	0.148	0.219	0.830	−11.503	9.229
Acculturative stress	0.059	0.024	2.426	0.015	−0.011	0.107
Job stress	0.737	0.288	2.555	0.011	0.172	1.302
Social support	−0.540	1.000	−0.540	0.589	−2.500	1.420
Perceived discrimination * Social support	0.005	0.028	0.175	0.861	−0.050	0.060
Model fit: Chi-square = 0.0304, *p* = 0.8616						

a Among the general characteristics, marital status and data collection period were adjusted as control variables.

b These results are expressed in a log-odds (logit) metric.

c * indicated the interaction effect.

d SE, standard error; LLCI, low limit confidence interval; ULCI, upper limit confidence interval.

## Discussion

This study investigated the moderating effect of social support on the relationship of acculturative stress, job stress, and perceived discrimination to depression among migrant workers living in South Korea during the COVID-19 pandemic. As hypothesized, acculturative stress was found to be a significant factor associated with depression of migrant workers, and social support showed a moderating effect. The most important finding by the Johnson–Neyman technique was that social support mitigated the effect of acculturation stress on depression only in the low social support group. This indicates that migrant workers with social support below a certain threshold should be prioritized for planning an intervention to prevent depression.

Our findings are consistent with prior research, which found the moderating effect of social support on negative health status (discrimination, low psychological well-being, and depression) due to acculturative stress [14, 36–38]. Our finding is consistent with the study of Fang et al. [35], showing that only under conditions of low social support, the association between acculturative stress and depression was pronounced among Chinese male immigrant workers. According to the social support level by Zimet [27], in this study, the migrant workers with high social support showed a non-significant effect of acculturative stress on depression. Given that two-thirds of the participants had been in their host country less than 5 years, this study is significant in that it confirmed that appropriate social support plays a pivotal role in a situation in which cultural adaptation is difficult and social support is insufficient.

A previous study demonstrated that the social support of co-workers is a critical factor in buffering the association between job stress and physical and mental health [39]. Also, the social support attenuated the effect of perceived discrimination on depressive symptoms of immigrant populations in South Korea [40]. However, in this study, the interaction of job stress and perceived discrimination on social support had no significant effect on depression. It is not clear why the moderating effect of social support was not observed. One potential explanation could be that the effect of social support is insufficient to offset the effects of perceived discrimination and job stress levels, as addressed by Ajrouch et al. [41]. In this study, we included workers from the manufacturing and service industries, such as food service workers and store clerks, who were vulnerable to COVID-19 related stress [42]. In South Korea, the government announced an intensive social distancing policy in March 2020 to control the spread of COVID-19 [43]. With the gradual increase in COVID-19 cases, social distancing was strengthened from August 2020 to October 2020. To prevent sporadic infections in the local community by limiting close contacts, face-to-face visits to community welfare facilities, which had previously provided migrant workers informational and emotional support, were restricted. Thus, it is inferred that this situation may lead to increased acculturative stress and missed opportunities to receive social support outside of work. In conclusions, it is suggested that migrant workers with less social support should be a priority population for preventing and managing depression and that the moderating role of social support should be applied to new migrant workers experiencing a high level of acculturative stress.

Most studies administered to prevent or alleviate mental health concerns tend to focus on individual factors rather than organizational or group factors [44]. In the study of Anjara et al. [45], social connection and proper management of the organization in the workplace were associated with the positive quality of life and mental health. In addition, poor residential conditions were significantly associated with perceived stress among migrant populations in urban China [46]. In order not to underestimate the discovery of underlying workplace organization deficiencies in future studies, it should be further examined the influence of organizational factors at work on mental health of migrant workers and determined interventional factors that can moderate the relationship. Migrant workers may be unaware of the seriousness of infectious diseases due to a lack of health information in their native language and may miss opportunities to learn about protective measures [47]. In addition, new practices triggered by the COVID-19 pandemic, such as social distancing and virtual environments, act as a barrier to the acculturation of migrants and lead to high stress levels. Therefore, social support is crucial because a lack of information on the prevention and management of infectious diseases, miscommunication, misinformation, and social isolation can further threaten the mental health of migrant workers. A meta-analysis of 64 studies on the relationship between social support and mental health reported a strong association between social support and mental health among workers [48].

In the current pandemic environment, international migrant workers are inevitably vulnerable due to limited access to health information, and they often experience loneliness or isolation because of limited social interactions. We suggest a multidimensional approach including access to health information, community support, and occupational health for migrant workers with a global call to action to protect the health of migrant workers from the effects of COVID-19. Since mobile phones are a commonly used communication tool among migrant workers, social support through social networking services may effectively alleviate their loneliness, stress, and COVID-19-related fears that may cause depression. Availability of social networking services can alleviate loneliness and stress while increasing psychological well-being by providing migrants with a sense of belonging and contact with others [49, 50]. Since virtual social activities may buffer the negative impacts related to COVID-19 [51], it would be beneficial to establish e-health communication channels to provide information tailored to their needs to help migrant workers prevent social isolation and encourage acculturation in the host country as intervention strategies.

Our study had several limitations. First, we recruited migrant workers through non-probabilistic sampling; thus, our findings may not be generalizable to the entire population. Second, cross-sectional studies cannot infer the causal mechanisms between discrimination, acculturative stress, job stress, and depression, underscoring the need for a long-term longitudinal study to confirm the relationship between the variables. Third, in this study, work organizational factors affecting migrant workers’ acculturative stress, job stress, and discrimination during a pandemic were not investigated, so it is necessary to confirm the relationship between these factors in future research. Nonetheless, the findings in this study have great significance as an attempt to confirm the phenomenon of stress and mental health problems of migrant workers who are easily alienated from the study population.

### Conclusion

In the context of COVID-19 pandemic, foreign migrants would be more vulnerable groups with limited information and social interaction. This study provides evidence that social support can serve as a buffer against the effects of acculturative stress on depression in the migrant worker population. In other words, it showed that the influence of acculturative stress on depression was greater in the group with low social support than in the group with high social support. Therefore, future research should identify the correlates of low social support and differentiate intervention strategies for improving mental health according to the level of social support of migrants. A research design that confirms differences by nationality is also suggested to understand social support within a cultural context considering cultural values and norms of each ethnic community.
